# Sequelae of Premature Birth in Young Adults: Incidental Findings on Routine Brain MRI

**DOI:** 10.1007/s00062-020-00901-6

**Published:** 2020-04-14

**Authors:** Dennis M. Hedderich, Tobias Boeckh-Behrens, Josef G. Bäuml, Aurore Menegaux, Marcel Daamen, Claus Zimmer, Peter Bartmann, Lukas Scheef, Henning Boecker, Dieter Wolke, Christian Sorg, Judith E. Spiro

**Affiliations:** 1 TUM-NIC Neuroimaging Center, Munich, Germany; 2 Department of Neuroradiology, Klinikum rechts der Isar, Technical University of Munich, School of Medicine, Ismaninger Str. 22, 81675 Munich, Germany; 3 Functional Neuroimaging Group, Department of Radiology, University Hospital Bonn, Bonn, Germany; 4 Department of Neonatology, University Hospital Bonn, Bonn, Germany; 5 Department of Psychology, University of Warwick, Coventry, UK; 6 Warwick Medical School, University of Warwick, Coventry, UK; 7 Department of Psychiatry, Klinikum rechts der Isar, Technical University of Munich, School of Medicine, Munich, Germany; 8 Department of Radiology, University Hospital, LMU Munich, Munich, Germany

**Keywords:** Preterm birth, Diagnostic imaging, White matter diseases, Neuroradiology, Lateral ventricles

## Abstract

**Background and Purpose:**

Qualitative studies about the abnormalities appreciated on routine magnetic resonance imaging (MRI) sequences in prematurely born adults are lacking. This article aimed at filling this knowledge gap by (1) qualitatively describing routine imaging findings in prematurely born adults, (2) evaluating measures for routine image interpretation and (3) investigating the impact of perinatal variables related to premature birth.

**Methods:**

In this study two board-certified radiologists assessed T1-weighted and FLAIR-weighted images of 100 prematurely born adults born very preterm (VP <32 weeks) and/or at very low birth weight (VLBW <1500 g) and 106 controls born at full term (FT) (mean age 26.8 ± 0.7 years). The number of white matter lesions (WML) was counted according to localization. Lateral ventricle volume (LVV) was evaluated subjectively and by measurements of Evans’ index (EI) and frontal-occipital-horn ratio (FOHR). Freesurfer-based volumetry served as reference standard. Miscellaneous incidental findings were noted as free text.

**Results:**

The LVV was increased in 24.7% of VP/VLBW individuals and significantly larger than in FT controls. This was best identified by measurement of FOHR (AUC = 0.928). Ventricular enlargement was predicted by low gestational age (odds ratio: 0.71, 95% CI 0.51–0.98) and presence of neonatal intracranial hemorrhage (odds ratio: 0.26, 95% CI 0.07–0.92). The numbers of deep and periventricular WML were increased while subcortical WMLs were not.

**Conclusion:**

Enlargement of the LVV and deep and periventricular WMLs are typical sequelae of premature birth that can be appreciated on routine brain MRI. To increase sensitivity of abnormal LVV detection, measurement of FOHR seems feasible in clinical practice.

**Electronic supplementary material:**

The online version of this article (10.1007/s00062-020-00901-6) contains supplementary material, which is available to authorized users.

## Introduction

Premature birth, defined as birth earlier than 37 gestational weeks, is estimated to have a prevalence of 11% worldwide and survival of preterm infants is increasing [1–3]. Preterm birth represents the major cause of infant mortality in high-income countries with increasing severity for lower gestational age [4]. Especially individuals born very prematurely, i.e. those born very preterm (VP <32 gestational weeks) or with very low birth weight (VLBW <1500 g) are at increased risk for long-term morbidity, for example with respect to neurocognitive impairment and psychiatric disorders [5–7]. Thanks to advances in neonatology, survival rates have increased in particular for very prematurely born children in the last decades [8].

Premature birth has been shown to be associated with an increased risk of white matter injury (WMI), either by relatively rare hemorrhagic or more commonly by nonhemorrhagic complications [2]. Perinatal WMI covers a spectrum ranging from its most severe form, cystic periventricular leukomalacia (PVL), to more diffuse forms caused by disturbed pre-oligodendrocyte maturation and axon myelination [9–11]. Punctate white matter lesions (PWML), ventriculomegaly due to white matter dysmaturation and diffuse excessive high signal intensity (DEHSI) on FLAIR-weighted images have been proposed as correlates of non-cystic WMI in prematurely born infants [10–12]; however, DEHSI has been shown to be a highly subjective imaging feature, dependent on the time of scanning and without reliable associations to outcomes of neurodevelopment [12, 13]. The pathophysiology underlying long-term structural brain alterations is under extensive investigation on a microstructural and macrostructural level using advanced neuroimaging methods [2, 14–17]. Due to the increased prevalence of survivors of premature birth, these individuals will become more likely to present to radiology departments and most likely undergo unrelated MRI examinations of the brain.

To date, there is a paucity of data regarding typical MRI findings in prematurely born adults that can be appreciated on routine sequences; however, this information will help the correct interpretation of presumably incidental findings. The purpose of this study was to qualitatively describe incidental findings with an emphasis on FLAIR-hyperintense PWML and ventricular enlargement in a large cohort of prematurely born adults and controls born at full term (FT). These findings were correlated with parameters of premature birth and it was established how to best measure ventricular size in order to identify ventriculomegaly in young adults in clinical practice. The impact of perinatal variables of premature birth on these findings was investigated and practical implications of the results in different diagnostic scenarios for the radiologist are discussed.

## Material and Methods

### Study Participants and Study Design

The participants examined in this study are part of the Bavarian Longitudinal Study, a geographically defined, whole population sample of neonatal at-risk children and healthy full-term controls who were followed from birth until adulthood [18, 19]. All participants underwent an MRI scan of the brain at approximately 26 years of age for research purposes only. For detailed information about recruitment of study participants, please see Fig. S1. The following conditions led to exclusion of participants before the MRI examination: claustrophobia, inability to lie still during the MRI, cerebral palsy, medical instability, epilepsy, tinnitus, previous central nervous system (CNS) trauma or disease, presence of MRI critical implants, and strong visual impairment. After exclusion of images with motion artifacts, FLAIR and T1 sequences were available for 100 VP/VLBW and 106 FT individuals. Papers about structural and functional imaging findings of subsamples of the present cohort of VP/VLBW were published before [16, 20–26]. The MRI examinations took place at two sites: The Department of Neuroradiology, Klinikum rechts der Isar, Technical University of Munich (*n* = 145) and the Department of Radiology, University Hospital Bonn (*n* = 67).

### Birth-Related Variables

Gestational age (GA) was estimated from maternal reports on the first day of the last menstrual period and serial ultrasounds during pregnancy. In cases where the 2 measurements differed by more than 2 weeks, clinical assessment at birth with the Dubowitz method was applied [27]. Maternal age, birth weight (BW) and duration of hospitalization, and presence of intracranial hemorrhage, including the grade of periventricular hemorrhage (PVH) [28] were obtained from obstetric records.

### MRI Data Acquisition

The MRI examinations were performed at both sites on either a Philips Achieva 3T or a Philips Ingenia 3T (Philips Medical Systems, Best, The Netherlands) system using 8‑channel SENSE head-coils. Subject distribution among scanners and sequence specifications are given in Table S1. Across all scanners, sequence parameters were kept identical for T1 and FLAIR sequences. Scanners were checked regularly to provide optimal scanning conditions. The MRI physicists at the University Hospital Bonn and Klinikum rechts der Isar regularly scanned imaging phantoms, to ensure within-scanner signal stability over time. Signal-to-noise ratio (SNR) was not significantly different between scanners (one-way ANOVA with factor “scanner-ID” [Bonn 1, Bonn 2, Munich 1, Munich 2]; F (3182) = 1.84, *p* = 0.11). For FLAIR sequences fast spin echo acquisition was used based on the variable refocusing 35° flip angle sweep technique [29].

### MRI Data Analysis

**Fig. 1:**
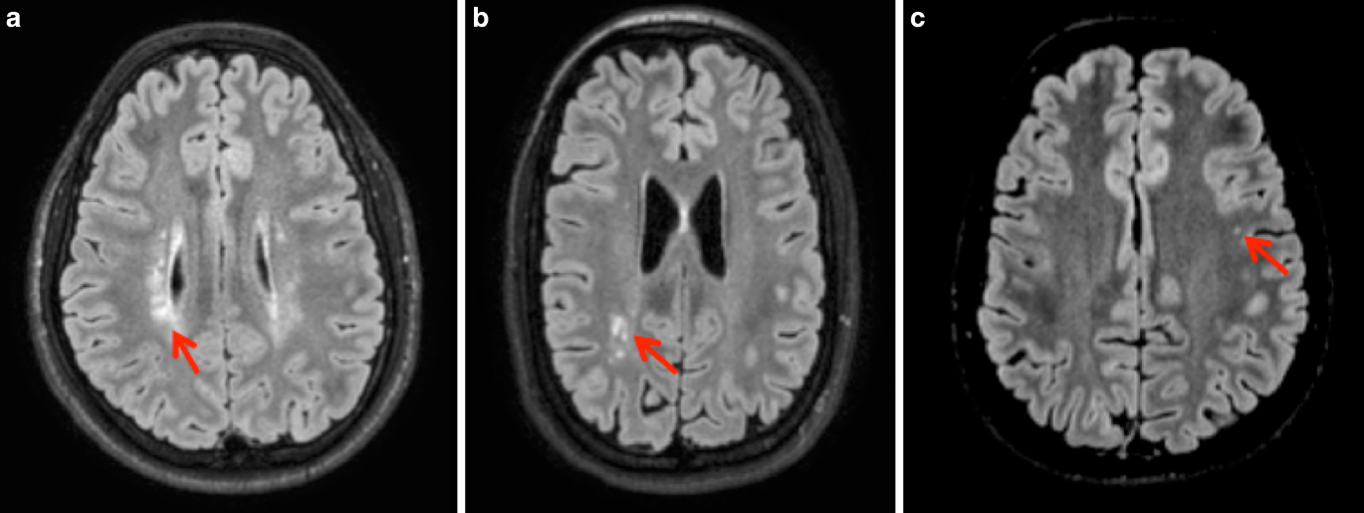
Fluid-attenuated inversion recovery (FLAIR) lesion localization. Examples of FLAIR hyperintense white matter lesions in periventricular (**a**), deep white matter (**b**) or subcortical (**c**) localization. Periventricular FLAIR lesions abut the ventricular surface, subcortical FLAIR lesions lie within 3 mm of the cortical ribbon, without direct contact. Representative lesions are marked with a *red* *arrow*

Two board-certified radiologists with 6 years experience evaluated FLAIR and T1-weighted images blinded to birth status. Supratentorial hyperintense lesions on FLAIR-weighted imaging were counted separately depending on their localization (periventricular, deep white matter, subcortical). Subcortical localization was defined as a distance <3 mm from the cerebral cortex, periventricular localization was defined by direct contact with the ventricular surface. Examples of periventricular, deep white matter and subcortical FLAIR hyperintense lesions are shown in Fig. 1. Lateral ventricle volume (LVV) was rated subjectively based on the radiologist’s impression in a yes or no fashion. Additionally, the Evans’ index (EI) and FOHR were measured as described previously [30]: the EI is defined as the ratio between the largest axial diameter of the frontal lateral ventricle horns and the biparietal diameter [31] and FOHR is defined as the sum of largest axial frontal horn and occipital horn diameter divided by double the biparietal diameter [30]. Due to the well-established excellent intrarater and interrater reliability [32], EI and FOHR were only measured by reader 1. Incidental findings were noted as free text.

In order to obtain fully automated and volumetric measures as a reference standard, T1-weighted scans of all participants were analyzed with the Freesurfer image analysis suite v6.0, which is documented and freely available for download online (http://surfer.nmr.mgh.harvard.edu) and volumes of subcortical structures were extracted [33]. The LVV was calculated as the sum of extracted left/right lateral ventricle and left/right inferior lateral ventricle volumes. The ratio between bilateral LVV and total intracranial volume (TIV) was calculated in order to correct for effects of different head sizes between individuals. Abnormal volume of the lateral ventricle was defined as two standard deviations above the bilateral LVV/TIV ratio in the control group. Youden’s index was calculated to determine the optimal cut-off for FOHR and EI.

### Statistical Analysis

Statistical differences regarding the patient groups were tested by Student’s t‑test (age, maternal age, gestational age, birth weight) and χ^2^-test (sex). Logistic regression analysis was used to determine predictors of abnormal LVV, linear regression analysis was used to determine predictors of absolute LVV. Pearson correlation analysis was used to investigate the relationship between PVH grade and LVV. Interrater reliability for categorical variables was tested using Cohen’s kappa. Intraclass correlation coefficients were calculated to assess interrater reliability regarding flair lesion count for consistency from a 2-way mixed model. Statistical computations were performed with the SPSS software package (SPSS Statistics for Mac, version 25.0; IBM, Armonk, NY, USA). Statistical significance was set at *p* < 0.05.

### Ethics Statement

The study was carried out in accordance with the Declaration of Helsinki and was approved by the local institutional review boards (#153/10). Written consent was obtained from all participants. All study participants received travel expenses and a small payment for participation.

## Results

### Study Cohort

Group demographic and clinical background variables are shown in Table 1. From 682 VP/VLBW neonates included in the initial study cohort, 260 participated in the psychological assessment at age 26 years and 101 underwent brain MRI (see flow diagram of study participants in Fig. S1). There were no significant differences between the VP/VLBW and FT group regarding age at scanning (*p* = 0.152), sex (*p* = 0.937), and maternal age (*p* = 0.868). By design, VP/VLBW subjects had significantly lower GA (*p* < 0.001) and lower BW (*p* < 0.001) and were hospitalized for a longer time after birth (*p* < 0.001).

**Table 1:** Demographic data and perinatal variables

	VP/VLBW (*n* = 100)			FT (*n* = 106)			*p* value
	M	SD	Range	M	SD	Range	
Sex (male/female)	57/43	–	–	61/45	–	–	0.937
Age (years)	26.7	±0.6	25.7–28.3	26.8	±0.7	25.5–28.9	0.152
GA (weeks)	30.5	±2.1	25–36	39.7	±1.1	37–42	**<0.001**
BW (g)	1328	±313	630–2070	3395	±448	2120–4670	**<0.001**
Hospitalization (days)	72.3	±26.6	24–170	7.0	±3.0	2–26	**<0.001**
Maternal age (years)	29.5	±4.7	16–41	29.4	±5.1	18–42	0.868

Statistical comparisons: sex, SES with χ^2^ statistics; age, GA, BW, Hospitalization, maternal age, IQ with two sample t‑tests

*BW* birth weight, *FT* full-term, *GA* gestational age, *M* Mean; maternal age, maternal age at birth, *SD* standard deviation, *VP/VLBW* very preterm and/or very low birthweight

Of 100 premature-born adults, 16 were diagnosed with intracranial hemorrhage (ICH) in the neonatal period (PVH grade 1: 5 (31.3%), grade 2: 7 (43.8%), grade 3: 3 (18.8%), grade 4: 1 (6.3%)).

### Increased Rate of Ventricular Enlargement After Premature Birth

Automated lateral ventricle volumetry was successful in 186 of 206 cases and showed increased volumes for premature-born individuals (VLBW/VP: 21.5 ± 13.9 ml; FT: 14.1 ± 6.3 ml; *p* < 0.001). The LVV was also higher in premature-born adults if sides were considered separately (left LVV: VLBW/VP: 11.3 ± 8.4 ml; FT: 7.3 ± 3.4 ml; *p* < 0.001; right LVV: 10.1 ± 5.9 ml; FT: 6.8 ± 3.3 ml; *p* < 0.001) and if LVV was divided by total intracranial volume (TIV) (LVV/TIV ratio: VLBW/VP: 1.3 ± 0.8%; FT: 0.8 ± 0.4%; *p* < 0.001). In VLBW/VP individuals, LVV of 22 out of 87 (25.3%) were 2 standard deviations above the mean LVV in controls normalized to TIV and thus considered abnormal. The rate of abnormal LVV was 5.1% (5 out of 99) for FT individuals (*p* < 0.001). Premature birth was associated with an increased risk of adult ventricular enlargement (OR: 6.25, 95% CI: 2.25–17.35), while sex was not a significant predictor of ventricular enlargement (OR: 1.08, 95% CI: 0.46–2.56). Binary logistic regression analyses in prematurely born adults revealed that both GA at birth and presence of PVH at birth were significant predictors of abnormal adult LV enlargement (GA: OR: 0.71, 95% CI: 0.51–0.98; presence of ICH: OR: 0.26, 95% CI: 0.07–0.92), while BW and sex were not significant (BW: OR: 1.00, 95% CI: 0.999–1.002; sex: OR: 1.31, 95% CI: 0.43–4.00). In prematurely born individuals who were diagnosed with PVH during the neonatal period, grading of PVH neither predicted absolute adult LVV (OR: −0.32, *p* = 0.267) nor abnormal LVV (OR: 0.18, 95% CI = 0.2–1.46).

Subjective classification of LVV was abnormal in 25 out of 100 (25.0%) prematurely born individuals and in 2 out of 106 (1.9%) control individuals for reader 1 and in 16 out of 100 (16.0%) prematurely born individuals and in 2 out of 106 (1.9%) control individuals for reader 2. Cross-table analysis with the volumetric definition of abnormal LVV revealed a sensitivity and specificity of 55.6% and 93.8% and a positive and negative predictive value of 0.60 and 0.93, respectively for reader 1 and a sensitivity and specificity of 44.4% and 96.9% and a positive and negative predictive value of 0.71 and 0.91, respectively for reader 2. Interrater reliability was moderate (κ = 0.578; *p* < 0.001).

**Fig. 2:**
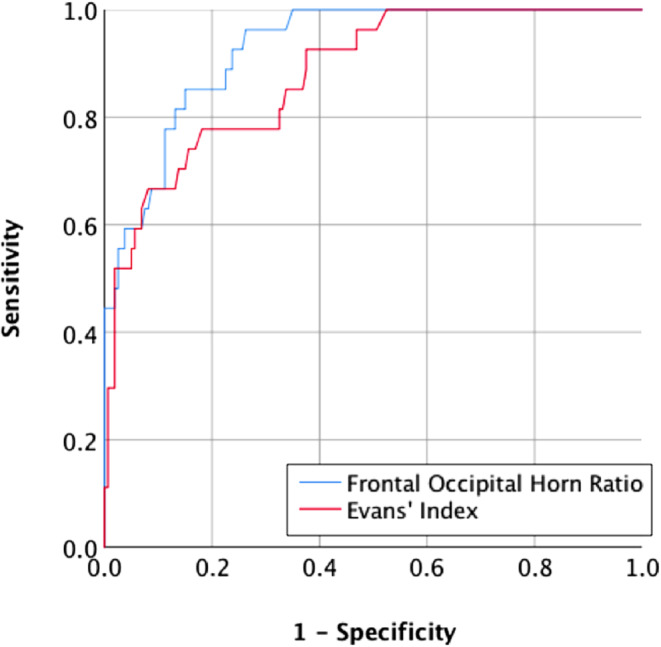
Accuracy of two-dimensional measurements of ventricular width. Receiver operating characteristics analysis showing the accuracy of Frontal Occipital Horn Ratio and Evans’ Index for classification of abnormal lateral ventricle volume

The ROC analysis revealed AUC of 0.928 (*p* < 0.001) and 0.880 (*p* < 0.001) for FOHR and EI, respectively. Youden’s J was calculated to identify the optimal cut-off for identifying abnormal LVV for both measures, leading to a value of 0.37 for FOHR and 0.27 for EI. These values were associated with sensitivities of 96.3% and 77.8% and specificities of 73.8% and 81.9% for FOHR and EI, respectively. The ROC curve is shown in Fig. 2.

### Prematurity is Associated with Deep White Matter Lesions on FLAIR Imaging in Adulthood

The FLAIR lesions were counted by two board-certified radiologists independently. Intraclass correlation coefficient (ICC) showed good to excellent agreement [34] (periventricular FLAIR lesions ICC = 0.945, 95% CI = 0.928–0.958; deep white matter FLAIR lesions ICC = 0.878, 95% CI =0.840–0.908; subcortical flair lesions ICC = 0.845, 95% CI = 0.796–0.882). All statistical analyses performed led to the same significant or insignificant results for reader 1 and 2. In the following, detailed numbers of the more experienced reader 1 are shown for reasons of simplicity and readability. A total of 115 individuals (55.8%) were positive for at least one FLAIR hyperintense WML in any location (VP/VLBW: 60/FT: 55; *p* = 0.259), 31 individuals (15.0%) were positive for periventricular FLAIR lesions (VP/VLBW: 24/FT: 7; *p* = 0.001), 49 individuals (23.8%) were positive for deep white matter FLAIR lesions (VP/VLBW: 33/FT: 16; *p* = 0.003) and 84 individuals (40.8%) were positive for subcortical FLAIR lesions (VP/VLBW: 35/FT: 49; *p* = 0.103). Detailed information on the distribution of periventricular, deep white matter, and subcortical FLAIR lesions can be found in Table 2, examples are shown in Fig. 1.

**Table 2:** Localization patterns of hyperintense FLAIR lesions in prematurely born and term-born adults

Localization	VP/VLBW			FT			*p*
	*n*	Median	IQR	*n*	Median	IQR	
Periventricular	24	2.0	[1.0–4.0]	7	1.0	[1.0–2.0]	0.053
DWM	33	4.0	[2.0–6.5]	16	1	[1.0–2.0]	*0.004*
Subcortical	35	3.0	[1.0–9.0]	49	2.0	[1.0–5.0]	0.447

Group differences of lesion numbers per localization were tested by Mann-Whitney U‑test. Cases without lesions in one of the three categories were not analyzed

*DWM* deep white matter, *FT* full-term, *VP/VLBW* very preterm and/or very low birthweight, *IQR* interquartile range

### Miscellaneous Incidental Findings

Pineal cysts >10 mm, with or without solid components needing follow-up examinations were found in 10 individuals (4.9%; 4 VP/VLBW; 6 FT); Tornwaldt cysts were seen in 4 cases (1.9%; 1 VP/VLBW; 3 FT) and 2 individuals showed a pituitary intermediate lobe cyst (1.0%; 1 VP/VLBW; 1 FT). Arachnoidal cysts were seen in two FT subjects in retrocerebellar and anterior temporal pole localizations. Partially empty sella was diagnosed in three subjects (1.4%; 1 VP/VLBW, 2 FT). One FT subject showed a cerebellar developmental venous anomaly. One FT subject was diagnosed with megacisterna magna. Ecchordosis physaliphora was diagnosed in one VP/VLBW patient. One VP/VLBW individual showed widening of the cerebellar foliae consistent with cerebellar hypoplasia without involvement of the vermis. One VP/VLBW subject was diagnosed with a Chiari I malformation and associated long-segment syringomyelia of the cervical spine.

Suspicious FLAIR hyperintense mass lesions were found in three subjects (1.4%; 2 VP/VLBW, 1 FT) located in the right and left pulvinar, and the left parietal lobe. One FT patient showed a 9 mm meningioma adjacent to the left occipital pole. A 9 mm subependymal mass in the lateral ventricle found in one VP/VLBW subject, suggesting disturbed neuronal migration.

## Discussion

This study qualitatively analyzed incidental findings in a large cohort of prematurely born adults and found increased rates of ventricular enlargement as well as an increased number of FLAIR hyperintense white matter lesions specifically located in the deep white matter. Significant predictors for abnormal lateral ventricle enlargement were low gestational age and history of periventricular hemorrhage in the neonatal period.

A previous study on a rather small subset of the present cohort investigated how well the neuroradiologist can distinguish between prematurely born adults and adults born at full-term [35]. They concluded that the discriminative power of visual inspection alone is suboptimal and can be increased by volumetric measurements. On the contrary, the present article aims at identifying characteristic incidental findings after premature birth in order to aid the radiologist at the correct classification of these imaging signs.

### Increased Lateral Ventricle Volume After Premature Birth

Ventriculomegaly is a mostly idiopathic and unspecific neuroradiological imaging finding potentially indicating either hydrocephalus or parenchymal atrophy. These two etiologies can be reliably distinguished by midsagittal morphological changes such as stretching and displacement of the corpus callosum, widening of the third ventricular recessus, and decreased mamillopontine distance [36]. We confirmed the expected low rate of ventricular enlargement in control individuals born at full-term; however, one out of four prematurely born adults exhibited enlargement of the LV, making this a common incidental finding after prematurity. In line with previous studies from prematurely born infants and adolescents, we demonstrated higher mean LVV in prematurely born adults [37, 38]. Larger LVV was significantly predicted by lower GA and incidence of PVH in the neonatal period, suggesting a parenchymal white matter deficit caused by either white matter dysmaturation and/or direct damage by ICH. Comparing three approaches regarding the evaluation of LV size in young adults, we have identified the FOHR as the most accurate measure being feasible in clinical routine [30]. This superior accuracy of the FOHR is most likely caused by occipital horn dilatation in prematurely born adults which was described previously [39].

### Increased FLAIR Hyperintense WMLs After Premature Birth in the Deep White Matter

Premature birth has been found to have an impact on the white matter either by causing direct damage and tissue necrosis or by inhibiting the development of pre-oligodendrocytes and subsequent altered axonal maturation [2, 10, 11]. This may result in visible or invisible alterations of the white matter on MRI ranging from cystic degeneration and parenchymal deficit to PWML and normal appearing white matter [10, 11]. The PWML are a common finding on brain MRI and are associated with increasing age and cardiovascular risk factors [40–42]. The presumed histopathological correlate of these PWML is astrogliosis following minor ischemia [43]. In the context of preterm birth, FLAIR hyperintense PWML reflect moderate WMI and have been described as a prognostic marker for adverse mental and psychomotor development in prematurely born infants at term-equivalent age [12]. One of the main findings of the study is that the total number of PWML is not increased in VP/VLBW adults; however, prematurely born adults were more likely to exhibit PWML in periventricular localization and in the deep white matter. This might indicate a pattern of prematurity affecting distinct white matter regions and reflect different underlying pathophysiological principles. Of course, besides particular vulnerability of the premature white matter towards hypoxic/ischemic events in the context of premature birth, other causes, such as infectious disease might lead to PWMLs visible on FLAIR imaging; however, the impact of severe CNS infections on the appearance of PWMLs can be considered small since a history of neurologic disease (including meningitis and encephalitis) served as exclusion criterion for MRI participation.

### Miscellaneous Findings in the Context of Premature Birth.

Despite the rather sizable study cohort, its size is too small to deduct frequencies of rare incidental findings. One prematurely born individual exhibited a subependymal remnant of neuronal tissue. This is likely related to premature birth, since the neuronal migration period extends well into the third trimester and thus coincides with preterm birth [44]. We observed overt cerebellar hypoplasia in one preterm individual. The cerebellum is known to be vulnerable in preterm neonates and impaired growth after premature birth has been previously shown [9]. Other incidental findings in the VP/VLBW cohort such as ecchordosis physaliphora or Chiari I malformation are very unlikely to be related to premature birth [45].

### Practical Considerations for the Radiologist

We have described increased rates of incidental findings on routine MRI after premature birth and think that this could have implications for radiological reporting in two distinct scenarios. Either the radiologist knows that a patient was born prematurely or it is unknown. If the radiologist knows, ventricular enlargement or increased periventricular or DWM punctate white matter lesion load can be interpreted as long-term sequelae of preterm birth and alternative explanations such as hypertension and/or chronic inflammatory CNS disease become less likely. If the radiologist is not aware of the patient’s birth history (which will be the case most of the times), findings such as ventricular enlargement or a certain distribution of PWMLs in periventricular or deep white matter locations should prompt thorough history taking before making a diagnosis.

### Strengths and Limitations

Some points should be carefully considered when interpreting the results. The current sample is biased to VP/VLBW adults with less severe neonatal complications. Individuals with more severe birth complications and/or severe lasting impairments in the initial BLS sample were more likely to drop out of this study covering more than two and a half decades from the neonatal period until adulthood and to be excluded in initial screening for MRI due to exclusion criteria for MRI. This selection towards less serious cases should be kept in mind when interpreting the results.

Unfortunately, no structured physical examinations were performed at the 26-year follow-up visit, so that direct information about clinically evident findings is limited; however, due to the previously mentioned selection bias towards less severe cases and through the exclusion criteria for MRI participation as mentioned in the Material and Methods section (e.g. cerebral palsy, history of neurologic disease or CNS trauma), it seems justified that the analyzed imaging features in fact constitute incidental findings.

Unfortunately, no sequence to detect blood degradation products (T2* weighted or susceptibility weighted imaging) was included in the study protocol owing to the overall study design. We consider it a strength of our study that a relevant impact of patient age on MRI findings at the time of the scan is excluded because all participants had approximately the same age of 26 years.

## Conclusion

Structural brain abnormalities visible on routine brain MRI sequences are commonly found in prematurely born adults. Prematurity at birth, in particular low GA and history of PVH is associated with ventricular enlargement, which could be best assessed by measuring the FOHR. The total number of PWML was not increased in prematurely born adults, however, the increased rate of deep white matter PWML suggested spatially distinct vulnerability of the white matter after premature birth.

## Caption Electronic Supplementary Material

Flow chart diagram of study participants and MRI sequence parameter settings.

## Abbreviations

AUC: Area under the curve
BW: Birth weight
CI: Confidence interval
EI: Evans’ index
FLAIR: Fluid-attenuated inversion recovery
FOHR: Frontal-occipital-horn ratio
FT: Full term
GA: Gestational age
ICC: Intraclass correlation coefficient
ICH: Intracranial hemorrhage
LV: Lateral ventricle
LVV: Lateral ventricle volume
OR: Odds ratio
PVH: Periventricular hemorrhage
PVL: Periventricular leukomalacia
PWML: Punctate white matter lesions
SNR: Signal-to-noise ratio
TIV: Total intracranial volume
VLBW: Very low birth weight
VP: Very preterm
WMI: White matter injury
WML: White matter lesion
