# Supplementary material for: Sequelae of Premature Birth in Young Adults: Incidental Findings on Routine Brain MRI
Source: Clin Neuroradiol. 2020 Apr 14;31(2):325–33. doi: 10.1007/s00062-020-00901-6 (PMC8211575; doi:10.1007/s00062-020-00901-6)
Supplement: Supplementary file 1 — Flow chart diagram of study participants and MRI sequence parameter settings. [file 62_2020_901_MOESM1_ESM.docx]

**Supplemental information**

Figure S1


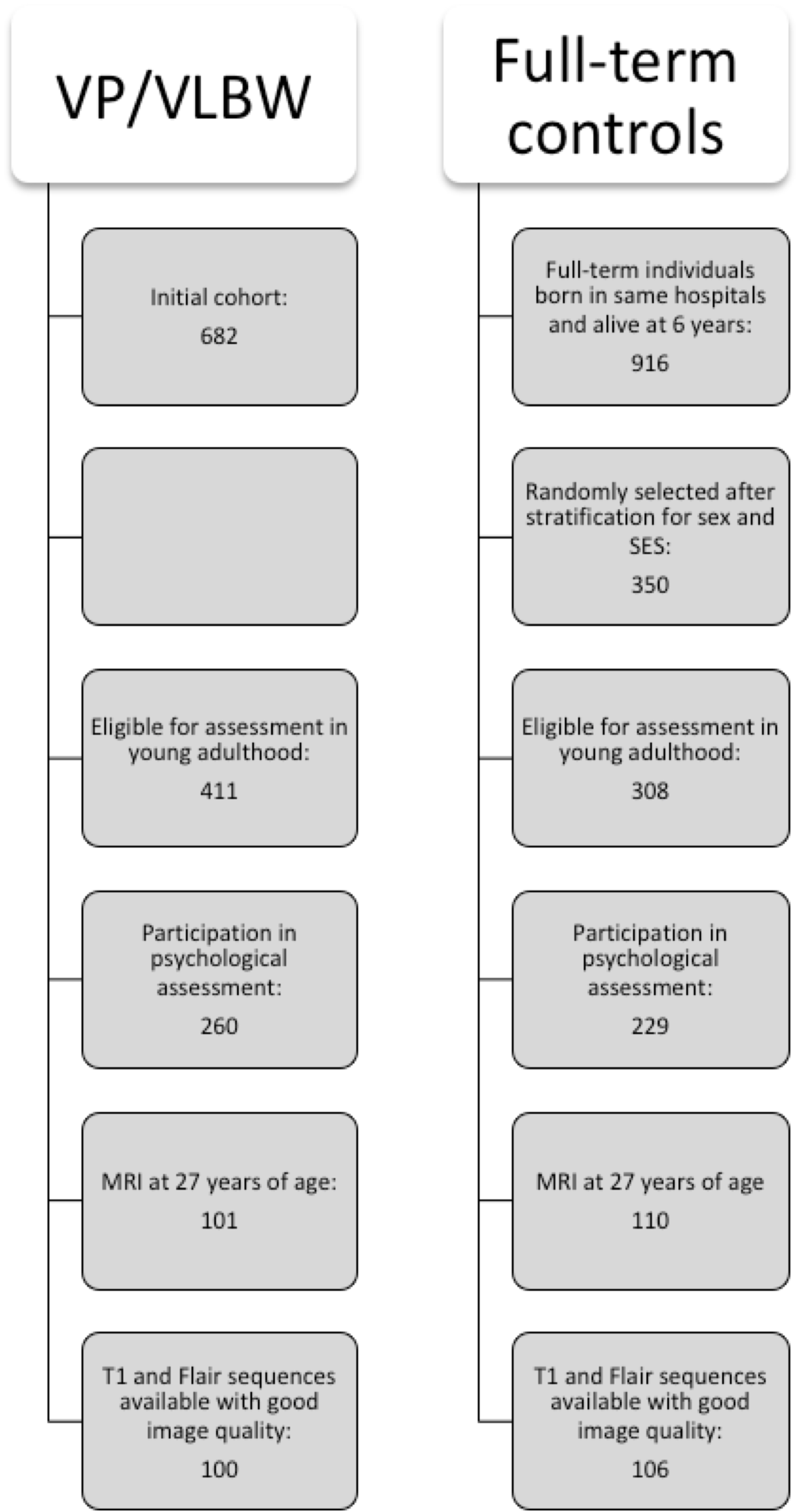


Figure S1: Flow diagram of study participants.

Abbreviations: SES: socioeconomic status, VP/VLBW: very preterm and/or very low birth weight

Table S1: Scanner distribution and sequence specifications

|  | Center | Bonn | | Munich | |
| --- | --- | --- | --- | --- | --- |
|  | Scanner | Achieva 3T | Ingenia 3T | Achieva 3T | Ingenia 3T |
|  | Subjects (VP/VLBW/ FT) | 5 / 10 | 33 / 17 | 60 / 64 | 2 / 15 |
| T1-weighted | TI | 1300 ms | 1300 ms | 1300 ms | 1300 ms |
|  | TR | 7.7 ms | 7.7 ms | 7.7 ms | 7.7 ms |
|  | TE | 3.9 ms | 3.9 ms | 3.9 ms | 3.9 ms |
|  | Flip angle | 15° | 15° | 15° | 15° |
|  | Field of view | 256 mm x 256 mm | 256 mm x 256 mm | 256 mm x 256 mm | 256 mm x 256 mm |
|  | Reconstructed voxel size | 1 mm^3^ | 1 mm^3^ | 1 mm^3^ | 1 mm^3^ |
| FLAIR-weighted | TI | 1600 ms | 1600 ms | 1600 ms | 1600 ms |
|  | TR | 4800 ms | 4800 ms | 4800 ms | 4800 ms |
|  | TE | 320 ms | 320 ms | 320 ms | 320 ms |
|  | Flip angle | 90° | 90° | 90° | 90° |
|  | Field of view | 256 mm x 256 mm | 256 mm x 256 mm | 256 mm x 256 mm | 256 mm x 256 mm |
|  | Reconstructed voxel size | 0.5 mm^3^ | 0.5 mm^3^ | 0.5 mm^3^ | 0.5 mm^3^ |

Table S1: Scanner distribution and sequence specifications.

Abbreviations: FLAIR: Fluid-attenuated inversion recovery; TE: Time to echo; TI: Time to inversion; TR: Time to repetition; VP/VLBW: very preterm and/or very low birth weight
